# Detection of Hepatitis B virus in serum and liver of chickens

**DOI:** 10.1186/1743-422X-9-2

**Published:** 2012-01-04

**Authors:** Jijing Tian, Kangkang Xia, Ruiping She, Wengui Li, Ye Ding, Jiande Wang, Mingyong Chen, Jun Yin

**Affiliations:** 1 Department of Veterinary Pathology, Key Laboratory of Zoonosis of Ministry of Agriculture, College of Veterinary Medicine, China Agricultural University, Beijing, 100193, China; 2 Beijing Huadu Broiler Corporation, Beijing 102211, China; 3 College of Animal Science and Technology, Yunnan Agricultural University, Kunming 650201, China

## Abstract

Hepatitis B virus (HBV) is one of the most important human pathogens. Its existence in food animals could present a significant threat to public health. The objective of this study was to determine if HBV is present in serum and liver of chickens. A total of 129 serum samples from broiler chickens were collected for the detection of HBV antigens and antibodies, and 193 liver samples were tested for HBV DNA sequence by PCR and for the existence of HBV antigens by immunohistochemistry. The overall prevalence of HBsAg, anti-HBs, anti-HBc was 28.68%, 53.49%, 17.05%, respectively, whereas HBeAg, anti-HBe were barely detectable. Three serum samples were found to be positive for both HBsAg and HBeAg. Further analysis of these samples with transmission electron microscopy (TEM) revealed two morphologic particles with 20 nm and 40 nm in diameter, which were similar to small spherical and Danes particles of HBV. The viral DNA sequence identified in two of the chicken livers shared 92.2% of one known HBV strain and 97.9% nucleotide sequence of another HBV strain. Our results showed the existence of HBV in chickens. This would present a significant risk to people who work with live chickens or chicken products if HBV found in chicken could be confirmed to be the same as human HBV.

## Background

Hepatitis B virus (HBV) is one of the most important human pathogens. More than 350 million people worldwide are persistently infected with HBV and are at risk of developing chronic liver disease, cirrhosis and hepatocellular carcinoma [1]. While vertical transmission of HBV from mother to neonate always results in chronic hepatitis, infection during adulthood results in lifelong protective immunity [2]. Although measures such as vaccination have been taken for years, the prevalence of HBV has not been controlled effectively, and it is still a major threat to human health.

The HBV genome is a relaxed circular DNA of ~ 3 200 nucleotides and consists of full length of the negative strand and a shorter positive strand. Serologic markers of hepatitis B virus infection include both viral antigens (surface antigen, HBsAg, and e antigen, HBeAg) and antibodies (anti-HBs, anti-HBc, anti-HBe). HBsAg is the most frequently used to screen for the presence of HBV infection. The presence of HBeAg in a host's serum is associated with much higher rates of viral replication and enhanced infectivity [3]. Detection of all the serologic markers is meaningful for clinical diagnosis of HBV in human.

Infection of HBV has already been documented in non-human primates (NHPs)[4] such as chimpanzees [5,6] and gorillas [7,8] in sub-Saharan Africa; gibbons and orangutans in South-East Africa [7,9]. Epidemiological studies have shown a high prevalence of HBV infection in great apes, that is comparable to human population in Gabon and Congo [7]. Furthermore, our team has found the existence of HBV in swine [10], indicating the possibility of HBV infection in food animals. Although there is currently no evidence that human population have been or are infected with food animal-associated HBV variants, existence of HBV in food animals deserves greater attention from researchers and the general public. Chickens are widely consumed by people all over the world, but it is not clear whether chickens have HBV infection. The objective of this project was to determine if HBV is present in chickens.

## Results

High percentages of the serum samples were found to be positive for HBsAg (28.68%, 37/129), anti-HBs (53.49%, 69/129) and anti-HBc (17.05%, 22/129), whereas HBeAg and anti-HBe was detected only in 4.65% (6/129) and 9.3% (12/129) of the samples respectivel. Only three of the 129 serum samples were positive for both HBsAg and HBeAg (Table 1).

**Table 1:** Detection of HBV Markers in Chicken Serum Samples

	Samples (n)	Positive Samples (n)	Positive Ratio (%)
HBsAg	129	37	28.68

anti-HBs	129	69	53.49

anti-HBc	129	22	17.05

HBeAg	129	6	4.65

anti-HBe	129	12	9.30

HBsAg + HBeAg	129	3	2.33

**Figure 1:**
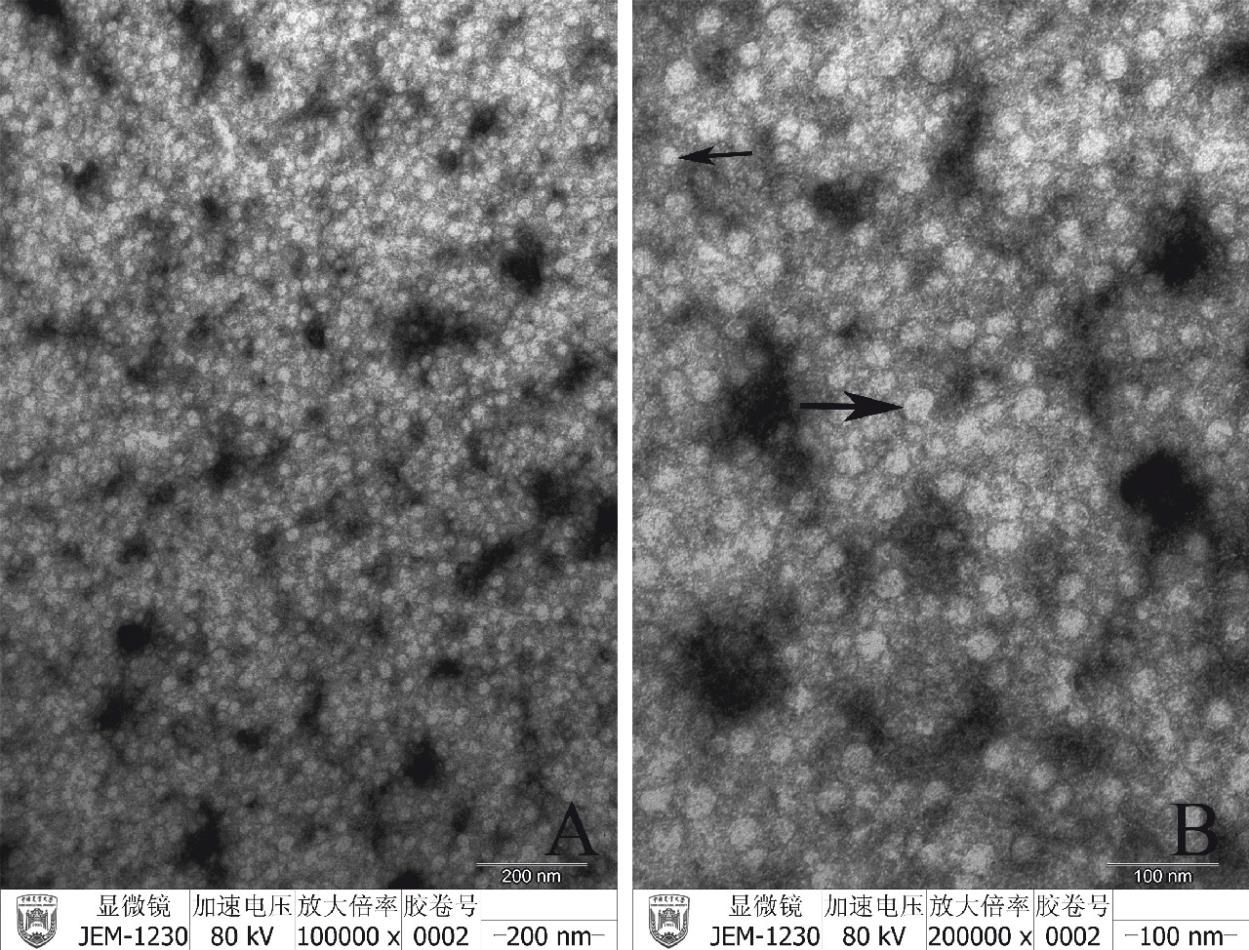
**Observation of hepatitis B virus like particles in chicken serum with TEM**. Arrows show HBV-like particles (A, Bar 200 nm; B, Bar = 100 nm).

Further analysis of these serum samples with TEM found that they contained two types of particles, the size and morphology of which were very similar to complete and empty viral particles of HBV (Figure 1). The one with a diameter of 40 nm appeared to be HBV Dane particle; and the other, with a diameter of 20 nm, was similar to small spherical particles of HBV in human serum.

**Figure 2:**
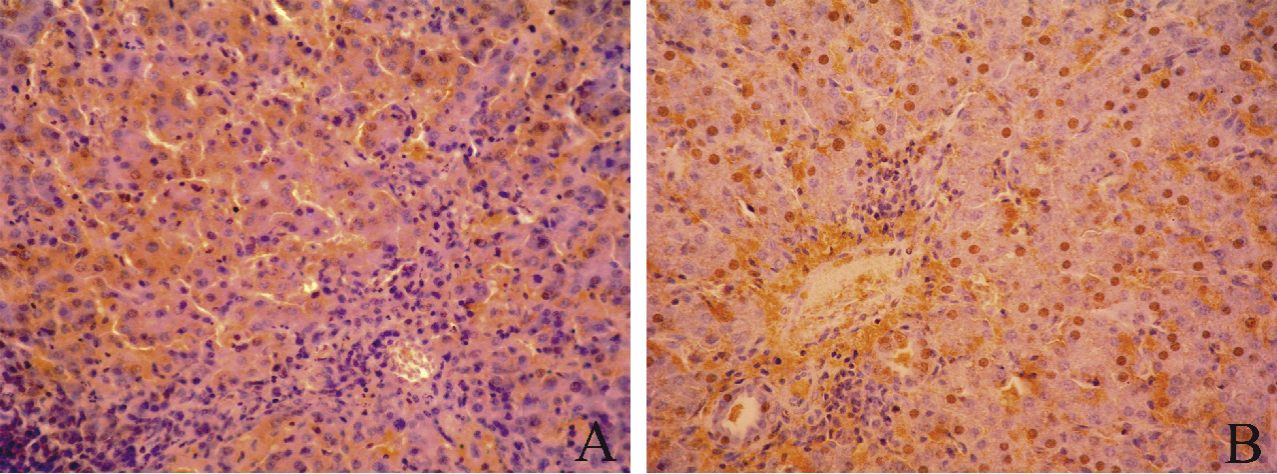
**Immunohistochemical analysis of HBsAg and HBcAg in liver tissues of chickens**. HBsAg was distributed mostly in cytoplasm of hepatocytes (A, 400×), and HBcAg was distributed mostly in the nucleus of hepatocytes (B, 400×).

Immunohistochemical staining showed that liver tissues from chickens were positive for HBsAg and HBcAg (Table 2). Under the microscope, HBsAg was detected in cytoplasm of hepatocytes, while HBcAg was mainly distributed in the nucleus of hepatocytes. In addition, a number of lymphocytes were found in the portal area and among hepatocytes, indicating that HBV was pathogenic to chickens, and replication of HBV might be responsible for the hepatitis lesions observed in the sections (Figure 2).

**Table 2:** Detection of HBsAg and HBcAg in Chicken Liver Samples By Immunohistochemical Staining (n = 193)

	HBsAg	HBcAg
Positive Samples (n)	106	86

Positive Ratio (%)	54.9	44.6

**Figure 3:**
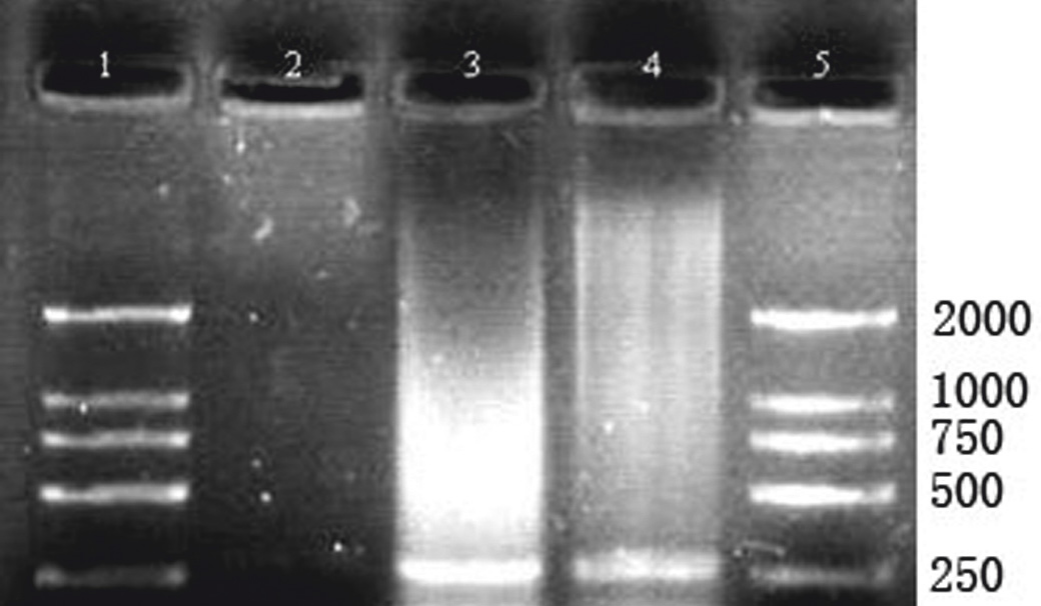
**Identification of the PCR products ofHBV in livers of chickens by agarose gel electrophoresis**. 1, 5: DL2000 DNA marker; 2: Negative control; 3, 4: Liver samples.

**Figure 4:**
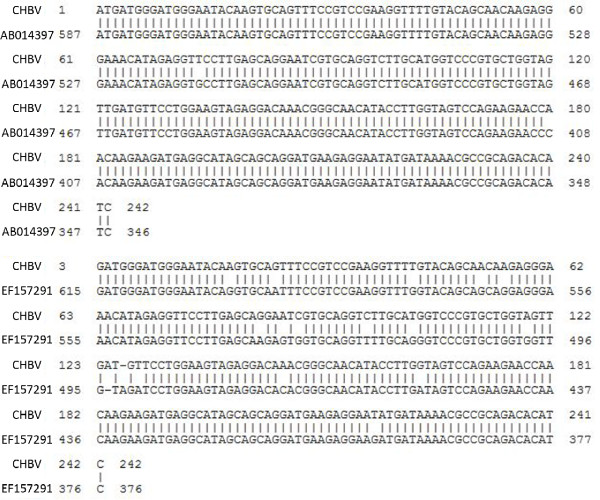
**Sequences of HBV in chickens and two related Human HBV strains**. DNA sequence of HBV in chickens shared 92.2% of human HBV strain EF157291 and 97.9% of the nucleotide sequence of AB014397.

Of the 193 liver samples, only two were found to contain HBV DNA (Figure 3) of the same sequence (Figure 4). This DNA shared 92.2% of the nucleotide sequence with the known HBV strain EF157291 (Hepatitis B virus isolate B3586-YKH94, complete genome, Japan)[11] and 97.9% of nucleotide sequence for the HBV strain AB014397 (Hepatitis B virus genomic DNA, complete sequence, isolate 38Y20HCC, Japan)[12], respectively (Table 3).

**Table 3:** Comparison of Amplified Sequences from the HBV in Chickens with That from HBV EF157290 and AB014397*

**Strain No**.	ChickenHBV		EF157290	AB014397
ChickenHBV		100.0	92.2	97.9
	
	0.0		92.2	97.9

EF157290	8.3	8.3		91.5

AB014397	2.2	2.2	9.1	

*The identities (%) are in the right-up part of the table, and the divergences (%) are in the left-down part of the table.

## Discussion

The present study is the first to report high prevalence of HBV infection in chickens, as indicated by the findings that 28. 68% and 53.49% of the chicken serum samples were positive for HBsAg and anti-HBs, and 54.9% and 44.6% of the liver samples were positive for HBsAg and HBcAg (Table 1, Table 2). Hepatitis B virus has been reported in other mammals (orthohepadnaviruses) and birds (avihepadnaviruses). Avihepadnaviruses have been reported in various duck species (WMHBV), grey herons (HHBV), geese (GHBV), Ross's goose (RGHBV), storks (STHBV), and cranes (CHBV)[13-15]. However, there were no reports of chicken HBV in the literature. How did the chickens get infected by HBV is unknown. Duck hepatitis B virus was the first of its kind to utilize an avian host where it was found in the bloodstream of an egg-laying duck. The virus was passed on from the infected duck to the egg resulting in congenital infection [16]. It is conceivable that the same could happen with HBV in chickens. The fact that different HB antigens and antibodies were detected in some of the chicken serum and liver samples but not in others might reflect different stages of HBV infections in the chickens. Interestingly, two types of viral particles, found in serum samples positive for both HBsAg and HBeAg, were similar to Dane particle and small spherical particle of human HBV in size and morphology, providing morphological evidence of HBV infection in chickens.

Immunohistochemistry staining is a common method for HBV detection [17]. Others reported that the marker and intensity of hepatocytes staining positive for HBsAg, as well as the cellular pattern of distribution, were related to HBV replication in patients with HBeAg-positive chronic hepatitis B (CHB). In general, HBsAg was located in the cytoplasm, whereas HBcAg was predominantly located in the nucleus in livers of HBV infection [18,19]. In this study, positive signals of HBsAg and HBcAg were both observed in liver specimens from chickens. Distribution pattern of the two antigens was the same as observed in HBV patients. Moreover, inflammatory signs observed in some of the liver samples such as accumulation of lymphocytes in the portal area and among hepatocytes suggest that HBV infection could lead to pathological changes in the liver of chickens. However, prevalence of HBsAg and HBcAg in liver was much higher than HBV DNA detected by PCR method. We found the same phenomenon in HEV (hepatitis E virus) infection in swine and chicken (data not show).

Alhough hepadnaviruses are usually host specific, HBV infections also occur frequently in chimpanzee, gorillas, gibbon and other ape populations in sub-Saharan Africa and South-east Asia where the HBV infection rate in apes was remarkably comparable to that of human population in these areas [5-9]. Scientists are concerned about the ability of HBV to cross species barriers. PCR detection in this study confirmed the existence of HBV in liver tissues of chickens. Although HBV DNA was detected in only two of the 193 liver samples, the DNA sequences from the two samples were identical, indicating the same HBV strain might be responsible for the HBV infection in chickens. It was not surprising that why HBV DNA was only detected in two liver samples that were positive for HBsAg and HBcAg by immunochemistry method, because HBV was only detectable during the incubation period and many of the chickens probably had passed that period.

Most HBV infection in human can be traced to neonatal transmission, drug-injection, sexual activity, or occupational exposure. Other, causes of infection, less frequent, include household contact, hemodialysis, transmission from a surgeon [20], and a receipt of organ or blood products. However, for more than 20%-30% of patients, no clear risk factors could be identified. The high homology of DNA sequence of HBV from chicken with the known human HBV strains is of concern from a public health point of view, because it raises the possibility that the HBV found in chicken could be the same or a variant of HBV that is responsible for hepatitis B in human. Our team has reported the presence of HBV in another food animal, the swine [10]. It is very common that people come into contact with food animals such as swine and chickens or food animal products. If chickens were infected by HBV, chicken meat could become a source of infection for people who work with it, especially when they have accidental cuts in their hands. However, it is premature to speculate that HBV infection in food animals such as chicken might contribute to the spread of HBV among human populations. Further research is needed to confirm how common HBV infection is in chickens and whether HBV can be passed from people to chickens and vice versa.

## Conclusions

In conclusion, high prevalence of HBV antigens and antibodies was found in chicken serum and liver samples, indicating HBV infection in chickens. If the HBV found in chicken could be confirmed to be the same as human HBV, HBV infection in chicken would represent a very significant risk to people who work with chickens or chicken products.

## Methods

Broiler chickens (42 days old) were processed in a slaughter house in Beijing, following the standard "Chicken Slaughtering Operation Procedures GB/T 19478-2004". Blood was drawn from the jugular vein immediately after the chickens were stunned by an electric shock. Serum was collected after blood samples were allowed to coagulate and centrifuged, and was kept frozen at -20°C until analysis. All serum samples were screened for hepatitis B serological markers (anti-HBc, HBsAg, anti-HBs, HBeAg, and anti-HBe) with respective enzyme-linked immunosorbent assay (ELISA) kits (SIIC Kinghaw Biotech Co. Ltd., Beijing, China) according to the manufacturer's recommendations. The absorbance was read at 450 nm (Multiscan Titertek MCC). Blank, negative and positive controls were included on each plate.

To obtain ultrastructural evidence for the presence of HBV-related viral particles in chickens, the three serum samples found to be positive for both HBsAg and HBeAg, were centrifuged at 4000 rpm for 10 min, then 0.01 M poly ethylene glycol 6000 (PEG6000) was added into the subsequent upper aqueous phase. After incubation overnight at 4°C, the serum was centrifuged at 20,000 rpm for 1 h, resuspended in PBS and stained for 1 min with 1% uranyl acetate. For the thin section study, the fixative used was 2.5% paraformaldehyde-glutaraldehyde in 0.1 M cacodylate buffer (pH 7.4). The sections were postfixed in 1% OsO4 for 1 h, and treated with 1% uranyl acetate, dehydrated in ethanol and embedded in Epon 812. All electron micrographs were obtained with JEV1230 transmission electron microscope (JEOL Ltd., Tokyo, Japan) at 80 kV.

Liver samples were collected when chickens were eviscerated. A portion of each liver sample was fixed in 2.5% (v/v) glutaraldehyde-polyoxymethylene solution immediately with the rest frozen at -80°C for the detection of HBV DNA sequence. The fixed liver tissue samples were dehydrated and embedded in paraffin wax. Serial paraffin sections (4 μm) were prepared and kept at 37°C for more than 12 h. The sections were immersed in three consecutive washings in xylene for 5 min to remove paraffin, and then hydrated with five consecutive washings with alcohol in descending order 100, 95, 80, 70, 50% and deionized water respectively. Sections were incubated for 15 min and blocked with 3% peroxide at room temperature for endogenous peroxidase ablation. The following steps were carried out in a moist chamber. Sections were incubated with blocking buffer (Zymed Laboratories Inc., San Diego, USA) containing 20% normal goat serum (Gibco) and 80% PBS (0.01 M, pH 7.4) at room temperature for 30 min. After discarding the goat serum, sections were incubated in primary monoclonal antibodies against HBsAg and HBcAg (Zhongshan Golden Bridge Biotech Co. Ltd., Beijing, China) diluted in PBS, for 2 h at 37°C. After rinsing for 3 times in PBS-T, sections were incubated with the goat anti-mouse IgG conjugated with HRP (Sigma) at 37°C for 40 min and rinsed 3 times in PBS-T. The specimens were incubated with 3,3-diaminobenzidin (DAB; Zymed Laboratories Inc) at room temperature for 10 min in the dark. Finally, sections were stained with hematoxylin for 5 min after rinsing for 3 times in PBS-T, dehydrated, and mounted with neutral gums. Sections for the negative control group were prepared by the same steps as described above but with the HBsAg and HBcAg antibodies replaced by PBS.

Liver tissues (50 mg) were homogenized in 450 μl of Tris/NaCl/EDTA. After addition of NaDodSO_4 _(Sodium dodecyl sulfate) and proteinase K to a final concentration of 1% (wt/vol) and 1 mg/ml, respectively, the homogenates were incubated for 24 h at 42 ~ 48°C before they were extracted with a phenol- chloroform- isoamylol, 25:24:1 (vol/vol) mixture. The DNA was precipitated by adding 1/10 vol of 3 M NaOAc and 2 vol of 100% EtOH. After being centrifuged and washed with 70% ice-cold ethanol, the DNA was dried under vacuum at room temperature. It was redissolved in TE buffer and store at -20°C.

Two primers [21] were used to detect genetically divergent strains of HBV (Table 4). Amplification conditions for PCR was: 30 cycles of 94°C for 4 min; 94°C for 30 s, 58°C for 30 s, 72°C for 40 s; 72°C for 5 min. Negative (water) control was included in each detection to exclude the possibility of contamination and failure of amplification. PCR products were sequenced by BGI (Beijing Genomics Institute, China). The sequences were compared to two known Japan HBV strains [11,12] in the GenBank database over the Internet by using the NCBI BLAST server [22].

**Table 4:** Primers Used in Detection of Hepatitis B Virus in Chicken Livers

	Sequence	Location	Product Size
**HBVs-F1**	5'-GAT GTG TCT GCG GCG TTT TA-3'	S gene	281 bp
		
**HBVs-R1**	5'- TTTTTCACCTCTGCCTAATCA-3'		

## Competing interests

The authors declare that they have no competing interests.

## Authors' contributions

JJT carried out the immunohistochemical staining and drafted the manuscript. KKX carried out the serological analysis of hepatitis B virus markers and PCR detection. WGL carried out the homology analysis. YD and MYC completed the transmission electron microscope investigations. JDW and JY did the pretreatment of animal samples. RPS carried out the design of the study and revision of the manuscript. JJT and KKX are joint first authors of this paper. All authors read and approved the final manuscript.
